# Study on the relationship between berry, grape, red wine consumption and cognitive impairment in middle-aged and elderly people in China

**DOI:** 10.3389/fnut.2024.1403427

**Published:** 2024-07-10

**Authors:** Xinting Jiang, Meirong Chen, Liang Cui, Qihao Guo, Lin Huang

**Affiliations:** ^1^Department of Gerontology, Shanghai Sixth People’s Hospital Affiliated to Shanghai Jiao Tong University School of Medicine, Shanghai, China; ^2^Department of Neurorehabilitation High Dependency Unit, Jiangwan Hospital, Shanghai, China

**Keywords:** cognitive impairment, grape, berry, red wine, anthocyanin, resveratrol

## Abstract

**Introduction:**

Some evidence suggests that fruit and alcohol consumption may be related to cognitive impairment.

**Methods:**

This study conducted a cross-sectional study on the “correlation between eating habits and cognitive function of the middle-aged and elderly population in China.” The purpose of this study is to explore the relationship between fruit consumption, drinking habits and cognitive impairment in Chinese people over 50 years old.

**Results:**

The results show that the protective factors of cognitive impairment are the preference for berries and the daily intake of 100-200 grams grapes in Chinese middle-aged and elderly people with objective cognitive unimpaired. The habit of drinking red wine is a protective factor for cognitive impairment in Chinese middle-aged and elderly people with mild cognitive impairment (MCI). However, this study did not find the relationship between white wine, beer, yellow rice wine, liquor and cognitive impairment.

**Discussion:**

Therefore, we believe that berries, grapes and red wine consumption can protect the cognitive function of the middle-aged and elderly people in China, and the protective function is related to the basic cognitive state.

## 1 Introduction

### 1.1 Preface

With the rapid growth of the elderly population in China, the prevalence of dementia such as Alzheimer’s disease (AD) has also increased in the past few decades (1). Cognitive decline is currently believed to be the most common cause of dementia (2). The focus of clinical and medical research is on the diagnosis and treatment of early clinical cognitive impairment (3). Recent studies have found that healthy eating patterns are associated with a lower risk of cognitive impairment among the elderly in China (4).

In recent years, studies have explored the relationship between berry, grape consumption and cognitive impairment. A systematic review of randomized controlled trials shows that berries and its supplements have beneficial effects on resting cerebral perfusion, cognitive function, memory performance, executive function, processing speed and attention index (5). Therefore, berry foods and their supplements may prevent cognitive decline in the elderly with cognitive health or mild cognitive impairment (MCI) (5). Studies have found that aged mice can improve the plasticity and memory ability of their brains by eating concentrated polyphenols extracted from grapes and blueberries for 14 weeks (6). Previous studies have also found that drinking blueberry or grape juice rich in polyphenols for 12 weeks can improve the cognitive ability of elderly subjects with normal cognition (7, 8).

Excessive drinking can lead to alcohol-related brain damage. In the 2020 dementia prevention guide published by the Lancet Committee, excessive drinking has been newly recognized as an important risk factor for dementia (9). However, there is controversy about the relationship between moderate drinking and neurodegenerative diseases in current research (10). A prospective cohort study found that non-drinking in people with normal cognition and drinking more than 14 drinks a week in people with MCI were associated with poor cognitive function compared with one drink per week (11). This study suggests that the association between alcohol consumption and dementia risk is related to baseline cognitive function and alcohol consumption (11). A study using large-scale data from National Alzheimer’s Coordinating Center (NACC) shows that there is a significant correlation between alcohol consumption and AD and Apolipoprotein E (APOE) (12). The study found that APOE gene carrier aggravates AD, while moderate alcohol intake has a potential positive effect on AD (12). The types of drinking and fruit consumption in China are different from those in Europe and the United States, so it is necessary to analyze the drinking habits and fruit consumption of Chinese people. Therefore, this paper explores the correlation between fruit consumption, drinking habits and cognitive impairment in people with different cognitive states.

### 1.2 Population study

This population-based cross-sectional study recruited those aged over 50 who took cognitive assessment in the Shanghai Sixth People’s Hospital Affiliated to Shanghai Jiao Tong University School of Medicine (China) from July 2019 to January 2023. The inclusion criteria involved participants who: (I) were 50 years old and above, regardless of sex; (II) had completed the standardized examination and diagnosed the degree of cognitive impairment. Participants were excluded if they met one of the following criteria: (I) missing clinical data or lost follow-up; (II) have suffered from depression, schizophrenia and other mental diseases; (III) were unable to cooperate with the inspection for various reasons; (IV) have suffered from various secondary cognitive disorders; (V) were unable to be grouped according to the diagnostic criteria of cognitive impairment. According to the inclusion and exclusion criteria, 1,127 questionnaires on eating habits were finally collected. Then, the subjects were divided into four groups according to their cognitive function, including 252 in the AD group, 350 in the MCI group, 276 in the SCD group and 249 in the NC group. The project was approved by the Ethics Committee of the Shanghai Sixth People’s Hospital Affiliated to Shanghai Jiao Tong University School of Medicine (China). The study was performed in accordance with the principles of the Declaration of Helsinki (approval number 2019–041). All participants provided written informed consent to participate in the study.

The recruitment methods, inclusion and exclusion criteria and ethical review of participants in this study are the same as those in the previous two articles (13, 14). The difference is that, on the basis of the previous study, this research added 235 participants, thus we re-conducted a univariate analysis of the Baseline Characteristics of all the participants.

### 1.3 Data collection

Similarly in the previous article, the dietary habit questionnaire was used to collect the clinical data of the subjects, including demographic data, basic physical condition, drug history and personal history (smoking habits, drinking habits) (13, 14). The difference is that in this study, we collected the fruit and alcohol consumption of middle-aged and elderly people with different cognitive states.

In terms of fruit consumption, we collected daily fruit consumption (> 200grams/ days, 100-200grams/ days, < 100grams/ days, no), consumption of fruit juice (no, yes), like berries (yes, no), daily berry consumption (> 200grams/ days, 100-200grams/ days, < 100grams/ days), like grapes (yes, no), daily grape consumption (> 200grams/ days, 100-200grasm/ days, < 100grams/ days). With reference to the drinking habits of Chinese people, in terms of alcohol consumption, we have collected habits of drinking red wine (yes, no), drinking white wine (yes, no), drinking beer (yes, no), drinking yellow rice wine (yes, no) and drinking liquor (yes, no).

### 1.4 Assessments

All participants in the study received in-depth neuropsychological evaluation that covered a wide range of cognitive functions. We conducted medical history inquiry, physical examination, neuropsychological test, laboratory examination (genetic detection such as APOE) and imaging examination (brain MRI, Amyloid β-PET). The evaluation of participants’ cognitive function and the grouping of participants can refer to the previous two articles (13, 14).

### 1.5 Data analysis

The statistical methods used in this study are the same as those in the previous two articles (13, 14). Four groups of subjects were divided into three categories: the NC group and the SCD group in Category one, the MCI group and the AD group in Category two, the objective cognitive unimpaired group (the NC group + the SCD group) and the objective cognitive impairment group (the MCI group + the AD group) in Category three.

Chi-square test was used to compare the demographic data, basic physical condition, drug history, smoking habits, drinking habits, fruit and alcohol consumption of the above three categories. Similarly, the continuous variables were compared by independent sample T test. The cognitive function of Category two and three was evaluated by covariance analysis, with age and education level as covariables. Classified variables are expressed by frequency (%), and continuous variables are expressed by mean ± standard deviation (M ± SD). Multivariable logistic regression analysis was performed for the independent associations with cognitive level using the variables at *p* < 0.05 from the univariate analysis. Considering that the metabolic state of diabetes may change the function of functional components of fruits and alcohol, that is, diabetes affects the nutritional bioavailability, we also included diabetes in the regression model for analysis. We used different logistic regression models to examine the relationship between fruit and alcohol consumption and different cognitive levels. The data were analyzed using SPSS25.0 software, and the difference was statistically significant with *p* < 0.05. The difference is that, according to the results of univariate analysis, we conducted a multivariate analysis on the consumption of berries, grapes and red wine in this study.

## 2 Results

### 2.1 Baseline characteristics of the study population

Table 1 shows the results of univariate analysis. Compared with the NC group, the SCD group had more female (*p* = 0.021) and more hypertension (*p* = 0.023). Compared with the MCI group, the AD group had older age (*p* < 0.001), shorter education years (*p* < 0.001), less constipation (*p* = 0.020), less allergic history (*p* = 0.002) and less dieting to lose weight (p = 0.002). Compared with the objective cognitive unimpaired group, the objective cognitive impairment group had older age (*p* = 0.006), shorter education years (*p* < 0.001), more tooth loss (*p* = 0.039), less chronic periodontitis (*p* = 0.032), less dieting to lose weight (*p* = 0.020) and less allergic history (*p* = 0.014).

**Table 1:** Research sample description based on cognitive state and objective cognitive impairment.

	NC group	SCD group	*p* ^a^	MCI group	AD group	*p* ^a^	Objective cognitive unimpaired group	Objective cognitive impairment group	*p* ^a^
N	249	276		350	252		525	602	
Demographic data									
Age (year)	64.5±8.9	64.8±8.7	0.755	67.8±7.1	70.5±7.8	<0.001	64.6±8.8	68.9±7.5	0.006
Education	12.7±3.2	12.4±3.1	0.403	10.9±3.3	9.5±4.2	<0.001	12.6±3.2	10.3±3.7	<0.001
BMI (kg/m^2^)	23.5±3.7	23.3±3.6	0.614	23.5±3.0	23.1±3.0	0.055	23.6±3.2	23.3±3.1	0.299
Waist (cm)	84.7±12.8	85.8±9.2	0.265	85.1±11.5	85.9±9.3	0.398	85.5±10.3	85.6±10.1	0.363
Sex									
Male	89 (35.7)	73 (26.4)	0.021	110 (31.4)	94 (37.3)	0.133	162 (30.9)	204 (33.9)	0.279
Female	160 (64.3)	203 (73.6)		240 (68.6)	158 (62.7)		363 (69.1)	398 (66.1)	
Marital status									
Never married	4 (1.6)	4 (1.5)	0.977	4 (1.2)	1 (0.4)	0.466	8 (1.5)	5 (0.9)	0.29
Married	227 (91.9)	253 (93.0)		305 (90.2)	231 (93.9)		480 (92.5)	536 (91.8)	
Widowed	8 (3.2)	8 (2.9)		19 (5.6)	10 (4.1)		16 (3.1)	29 (5.0)	
Divorced	8 (3.2)	7 (2.6)		10 (3.0)	4 (1.6)		15 (2.9)	14 (2.4)	
First-degree relatives with AD									
Yes	43 (19.6)	60 (22.9)	0.385	54 (16.5)	45 (18.8)	0.478	101 (21.0)	98 (17.3)	0.123
No	176 (80.4)	202 (77.1)		274 (83.5)	195 (81.3)		379 (79.0)	469 (82.7)	
APOE ε4 allele carries									
Yes	43 (18.8)	50 (19.5)	0.85	89 (25.4)	114 (46.2)	<0.001	96 (18.4)	203 (34.1)	<0.001
No	186 (81.2)	207 (80.5)		261 (74.6)	133 (53.8)		426 (81.6)	393 (65.9)	
MoCA-B Scores	25.9±0.2	25.2±0.2	0.003	20.4±0.2	13.2±0.3	<0.001	24.7±0.2	18.4±0.2	<0.001
ACE-III Scores	83.2±0.8	80.2±0.7	0.005	67.0±1.1	51.0±1.2	<0.001	79.4±0.9	62.7±0.8	<0.001
Basic physical condition									
Tooth loss									
No tooth loss	78 (31.8)	57 (21.4)	0.065	82 (23.4)	59 (23.4)	0.054	135 (26.4)	128 (22.0)	0.039
>20 teeth	86 (35.1)	97 (36.5)		119 (34.0)	70 (27.8)		183 (35.8)	183 (31.4)	
10-19 teeth	34 (13.9)	42 (15.8)		52 (14.9)	48 (19.0)		76 (14.9)	100 (17.2)	
1-9 teeth	41 (16.7)	58 (21.8)		84 (24.0)	54 (21.4)		99 (19.4)	138 (23.7)	
No teeth	6 (2.4)	12 (21.8)		13 (3.7)	21 (8.3)		18 (3.5)	34 (5.8)	
Chronic periodontitis									
No	162 (66.1)	154 (57.7)	0.05	222 (65.1)	176 (71.8)	0.085	316 (61.7)	398 (67.9)	0.032
Yes	83 (33.9)	113 (42.3)		119 (34.9)	69 (28.2)		196 (38.3)	188 (32.1)	
Periodontitis years									
<5 years	36 (43.4)	42 (40.7)	0.742	54 (47.0)	32 (45.1)	0.955	82 (41.8)	86 (46.2)	0.649
5-10 years	16 (19.3)	27 (23.9)		24 (20.9)	16 (22.5)		43 (21.9)	40 (21.5)	
>10 years	31 (37.3)	40 (35.4)		37 (32.2)	23 (32.4)		71 (36.2)	60 (32.3)	
Dieting to lose weight									
Yes	16 (6.5)	18 (6.7)	0.941	19 (5.5)	2 (0.8)	0.002	34 (6.6)	21 (3.6)	0.02
No	230 (93.5)	252 (93.3)		326 (94.5)	244 (99.2)		482 (93.4)	570 (96.4)	
Diarrhea									
Yes	69 (27.9)	93 (34.3)	0.118	98 (28.2)	58 (23.3)	0.182	162 (31.3)	156 (26.1)	0.058
No	178 (72.1)	178 (65.7)		250 (71.8)	191 (76.7)		356 (68.7)	441 (73.9)	
Constipation									
2-3 times/WKD	22 (8.9)	39 (14.4)	0.126	40 (11.6)	17 (6.8)	0.02	61 (11.8)	57 (9.6)	0.067
1 time/WKD	10 (4.0)	15 (5.5)		22 (6.4)	26 (10.4)		25 (4.8)	48 (8.1)	
1 every2-4 weeks	24 (9.7)	31 (11.4)		35 (10.1)	15 (6.0)		55 (10.6)	50 (8.4)	
No	192 (77.4)	186 (68.6)		248 (71.9)	192 (76.8)		378 (72.8)	440 (73.9)	
Surgical history within one year									
Yes	30 (12.1)	31 (11.3)	0.757	40 (11.5)	20 (8.0)	0.169	61 (11.7)	60 (10.0)	0.374
No	217 (87.9)	244 (88.7)		309 (88.5)	229 (92.0)		461 (88.3)	538 (90.0)	
Hypertension									
Yes	80 (32.3)	114 (41.9)	0.023	125 (36.3)	98 (39.5)	0.431	194 (37.3)	223 (37.7)	0.901
No	168 (67.7)	158 (58.1)		219 (63.7)	150 (60.5)		326 (62.7)	369 (62.3)	
Diabetes									
Yes	26 (10.5)	36 (13.2)	0.334	45 (13.1)	39 (15.8)	0.352	62 (11.9)	84 (14.2)	0.26
No	222 (89.5)	236 (86.8)		299 (86.9)	208 (84.2)		458 (88.1)	507 (85.8)	
Allergic history									
Yes	56 (22.5)	72 (26.2)	0.326	79 (22.6)	31 (12.4)	0.002	128 (24.3)	110 (18.4)	0.014
No	193 (77.5)	203 (73.8)		270 (77.4)	218 (87.6)		396 (75.6)	488 (81.6)	
Drug history									
Metformin									
No	218 (89.3)	241 (90.3)	0.732	294 (88.6)	216 (87.8)	0.634	459 (89.8)	510 (88.2)	0.403
Yes	26 (10.7)	26 (9.7)		38 (11.4)	29 (11.8)		52 (10.2)	68 (11.8)	
Antibiotics									
Yes	33 (13.3)	44 (16.0)	0.375	46 (13.2)	25 (10.0)	0.237	77 (14.7)	71 (11.9)	0.167
No	216 (86.7)	231 (84.0)		302 (86.8)	224 (90.0)		447 (85.3)	526 (88.1)	
Cognitive-promoting drug use									
YES	2 (0.8)	10 (3.6)	0.03	13 (3.8)	54 (21.7)	<0.001	12 (2.3)	83 (13.9)	<0.001
No	247 (99.2)	265 (96.4)		333 (96.2)	195 (78.3)		512 (97.7)	516 (86.1)	
Smoking habit									
Smoking at present									
Yes	21 (55.3)	16 (72.7)	0.18	23 (59.0)	22 (61.1)	0.85	37 (61.7)	45 (60.0)	0.844
No	17 (44.7)	6 (27.3)		16 (41.0)	14 (38.9)		23 (38.3)	30 (40.0)	
Secondhand smoke environment									
Yes	199 (82.9)	202 (78.6)	0.223	63 (19.0)	39 (16.8)	0.5	401 (80.7)	461 (81.9)	0.617
No	41 (17.1)	55 (21.4)		268 (81.0)	193 (83.2)		96 (19.3)	102 (18.1)	
Your parents smoked before you were born									
No	147 (60.7)	154 (59.0)	0.719	210 (62.7)	136 (57.4)	0.201	301 (59.8)	346 (60.5)	0.828
Yes	95 (39.3)	107 (41.0)		125 (37.3)	101 (42.6)		202 (40.2)	226 (39.5)	
Smoke or not									
>20/Day	4 (1.6)	3 (1.1)	0.216	7 (2.0)	5 (2.0)	0.234	7 (1.3)	12 (2.0)	0.445
10-20/Day	2 (0.8)	5 (1.8)		8 (2.3)	3 (1.2)		7 (1.3)	11 (1.8)	
1-10/Day	16 (6.4)	8 (2.9)		11 (3.2)	15 (6.0)		24 (4.6)	26 (4.3)	
Quit smoking	26 (10.4)	13 (4.7)		30 (8.6)	30 (11.9)		39 (7.4)	60 (10.0)	
Not at all	201 (80.7)	246 (89.5)		291 (83.9)	199 (79.0)		447 (85.3)	490 (81.8)	
Drinking habits									
Drinking frequency									
Not at all	130 (52.8)	163 (60.8)	0.326	210 (61.2)	158 (64.8)	0.67	293 (57.0)	368 (62.7)	0.085
Sometimes	94 (38.2)	87 (32.5)		103 (30.0)	62 (25.4)		181 (35.2)	165 (28.1)	
1-3 times/WKD	7 (2.8)	6 (2.2)		9 (2.6)	7 (2.9)		13 (2.5)	16 (2.7)	
> 4 times/WKD	15 (6.1)	12 (4.5)		21 (6.1)	17 (7.0)		27 (5.3)	38 (6.5)	

a Continuous variables were analyzed using independent sample T test, whereas categorical variables (proportions) were analyzed using the chi-square test.

*p*, *p* value. NC, normal control; SCD, subjective cognitive decline; MCI, mild cognitive impairment; AD, Alzheimer’s disease; BMI, Body Mass Index; MoCA-B, Montreal Cognitive Assessment—Basic; ACE-III, The Addenbrook’s Cognitive Examination; WKD, Weekend.

As expected, the SCD, AD and objective cognitive impairment groups performed more poorly on all cognitive tests and used more cognitive-promoting drugs compared with the corresponding groups in the three categories. Similarly, the AD and objective cognitive impairment groups carried more APOE ε4 alleles compared with the corresponding groups.

There was no significant difference in “Body Mass Index (BMI) and Waist” among the groups, suggesting that the overall nutritional status was similar. “Whether the first-degree relatives with AD or not and marital status” were not related to the severity of cognitive impairment. The three categories did not differ with respect to basic physical condition (surgical history within one year, diabetes or diarrhea), drug history (metformin use, antibiotic use), smoking habits and drinking frequency.

### 2.2 Correlation between fruit consumption, drinking habits and cognitive impairment

#### 2.2.1 Comparison of fruit consumption and drinking habits

Table 2 shows the results of univariate analysis. In terms of fruit consumption and drinking habits, compared with the NC group, the SCD group consumed less berries every day (*p* = 0.017). Compared with the MCI group, the AD group consumed less fruit per day (*p* = 0.006), less like berries (*p* = 0.011) and less drinking red wine habits (*p* = 0.001).

**Table 2:** Comparison of fruit consumption and drinking habits based on Cognitive State and Objective Cognitive impairment.

	NC group	SCD group	*p* ^a^	MCI group	AD group	*p* ^a^	Objective cognitive unimpaired group	Objective cognitive impairment group	*p* ^a^
									
*N*	249	276		350	252		525	602	
Fruit consumption, Drinking habits									
Consumption of fruit									
>200grams/day	47 (19)	52 (19.3)	0.245	63 (18.2)	34 (13.7)	0.006	99 (19.1)	97 (16.3)	0.004
100-200grams/day	137 (55.5)	132 (48.9)		168 (48.4)	98 (39.4)		269 (52.0)	266 (44.6)	
<100grams/day	58 (23.5)	74 (27.4)		107 (30.8)	103 (41.4)		132 (25.5)	210 (35.2)	
NO	5 (2.0)	12 (4.4)		9 (2.6)	14 (5.6)		17 (3.3)	23 (3.9)	
Consumption of fruit juice									
No	224 (95.7)	253 (96.9)	0.473	317 (95.5)	225 (96.2)	0.696	477 (96.4)	542 (95.8)	0.615
Yes	10 (4.3)	8 (3.1)		15 (4.5)	9 (3.8)		18 (3.6)	24 (4.2)	
Like berries									
Yes	164 (66.4)	160 (60.2)	0.143	182 (52.6)	103 (42.0)	0.011	324 (63.2)	285 (48.2)	<0.001
No	83 (33.6)	106 (39.8)		164 (47.4)	142 (58.0)		189 (36.8)	306 (51.8)	
Berry consumption									
>200grams/day	9 (4.1)	21 (8.7)	0.017	22 (7.3)	7 (3.2)	0.103	30 (6.5)	29 (5.6)	0.100
100-200grams/day	75 (33.8)	58 (24.1)		72 (23.9)	49 (22.4)		133 (28.7)	121 (23.3)	
<100grams/day	138 (62.2)	161 (67.2)		207 (68.8)	163 (74.4)		300 (64.8)	370 (71.2)	
Like grapes									
Yes	208 (84.9)	228 (86.0)	0.715	266 (78.0)	184 (75.1)	0.411	436 (85.5)	450 (76.8)	<0.001
No	37 (15.1)	37 (14.0)		75 (22.0)	61 (24.9)		74 (14.5)	136 (23.2)	
Grape consumption									
>200grams/day	22 (9.6)	25 (9.6)	0.996	33 (10.0)	12 (5.2)	0.099	47 (9.6)	45 (8.0)	0.001
100-200grams/day	90 (39.5)	104 (39.8)		99 (30.1)	68 (29.4)		194 (39.7)	167 (29.8)	
<100grams/day	116 (50.9)	132 (50.6)		197 (59.9)	151 (65.4)		248 (50.7)	348 (62.1)	
Red wine									
Yes	76 (31.0)	75 (28.2)	0.484	84 (24.9)	32 (13.3)	0.001	151 (29.9)	116 (20.1)	<0.001
No	169 (69.0)	191 (71.8)		32 (75.1)	208 (86.7)		354 (70.1)	461 (79.9)	
White wine									
Yes	13 (5.3)	18 (6.8)	0.49	13 (3.9)	3 (1.3)	0.06	31 (6.1)	16 (2.8)	0.007
No	232 (94.7)	248 (93.2)		324 (96.1)	237 (98.8)		474 (93.9)	561 (97.2)	
Beer									
Yes	35 (14.3)	31 (11.7)	0.376	31 (9.2)	26 (10.8)	0.517	66 (13.1)	57 (9.9)	0.099
No	210 (85.7)	235 (88.3)		306 (90.8)	214 (89.2)		439 (86.9)	520 (90.1)	
Yellow rice wine									
Yes	30 (12.2)	26 (9.8)	0.372	44 (13.1)	29 (12.1)	0.729	56 (11.1)	73 (12.7)	0.429
No	215 (87.8)	240 (90.2)		293 (86.9)	211 (87.9)		449 (88.9)	504 (87.3)	
Liquor									
Yes	28 (11.4)	27 (10.2)	0.641	28 (8.3)	23 (9.6)	0.595	55 (10.9)	51 (8.8)	0.257
No	217 (88.6)	239 (89.8)		309 (91.7)	217 (90.4)		450 (89.1)	526 (91.2)	

a Categorical variables (proportions) were analyzed using the chi-square test.

*p*, *p* value. NC, normal control; SCD, subjective cognitive decline; MCI, mild cognitive impairment; AD, Alzheimer’s disease.

Compared with the objective cognitive unimpaired group, the objective cognitive impairment group consumed less fruit per day (*p* = 0.004), less like berries (*p* < 0.001), less like grapes (p < 0.001), less grape consumption per day (*p* = 0.001), less drinking red wine (*p* < 0.001) and less drinking white wine habits (*p* = 0.007).

#### 2.2.2 Binary logistic regression analysis of the relationship between grape, berry, red wine consumption and cognitive impairment

Table 3 shows the results of Binary Logistic Regression analysis. Those who like berries in the objective cognitive unimpaired (compared with those who do not like berries) have a lower risk of cognitive impairment (OR = 0.543，95%CI, 0.427–0.692, *p* < 0.001).After adjusting for age, education, tooth loss, chronic periodontitis, dieting to lose weight, diabetes, allergic history, cognitive-promoting drug use in model b, this relationship is still statistically significant (OR = 0.640, 95%CI, 0.485–0.843, *p* = 0.002). On the basis of model b, consumption of fruit, like grapes, grape consumption, red wine, white wine, and APOE genotype carrying factors in model c did not change the results (OR = 0.685, 95%CI, 0.502–0.935, *p* = 0.017).

**Table 3:** Multivariate analysis of like berries.

Group		Model	Reference group		B	SE	Wald	*p*	OR	95%CI
X	Like berries									
		a	NO	YES	−0.610	0.123	24.562	<0.001	0.543	0.427–0.692
		b	NO	YES	−0.447	0.141	10.052	0.002	0.640	0.485–0.843
		c	NO	YES	−0.378	0.159	5.687	0.017	0.685	0.502–0.935

X, The objective cognitive unimpaired group and the objective cognitive impairment group. a, basic model, no adjustment. b, adjusted for age, education, tooth loss, chronic periodontitis, dieting to lose weight, diabetes, allergic history, cognitive-promoting drug use. c, adjusted for all variables in model b + consumption of fruit, like grapes, grape consumption, red wine, white wine, APOE genotype. B, Coefficient for the constant; SE, Standard error around the coefficient for the constant; p, p-value; OR, Exp (B); 95%CI, Confidence interval for the odds ratio with its upper and lower limits.

Table 4 shows the results of Binary Logistic Regression analysis. In the objective cognitive unimpaired group, those who take 100-200grams grapes per day (compared with those who take <100 g grapes per day) have a lower risk of cognitive impairment (OR = 0.613, 95%CI, 0.471–0.798, *p* < 0.001). After adjusting for age, education, tooth loss, chronic periodontitis, dieting to lose weight, diabetes, allergic history, cognitive-promoting drug use in model b, this relationship is still statistically significant (OR = 0.677, 95%CI, 0.499–0.917, *p* = 0.012). On the basis of model b, consumption of fruit, red wine, white wine and APOE genotype carrying factors in model c have not changed the results (OR = 0.702, 95%CI, 0.509–0.969, *p* = 0.031).

**Table 4:** Multivariate analysis of daily grape consumption.

Group		Model	Reference group		B	SE	Wald	*p*	OR	95%CI
X	Grape consumption									
		a	<100 g/d	100-200 g/d	−0.489	0.134	13.229	<0.001	0.613	0.471–0.798
		b	<100 g/d	100-200 g/d	−0.390	0.155	6.326	0.012	0.677	0.499–0.917
		c	<100 g/d	100-200 g/d	−0.353	0.164	4.638	0.031	0.702	0.509–0.969

X, The objective cognitive unimpaired group and the objective cognitive impairment group. a, basic model, no adjustment. b, adjusted for age, education, tooth loss, chronic periodontitis, dieting to lose weight, diabetes, allergic history, cognitive-promoting drug use. c, adjusted for all variables in model b + consumption of fruit, red wine, white wine, APOE genotype. B, Coefficient for the constant; SE, Standard error around the coefficient for the constant; p, p-value; OR, Exp (B); 95%CI, Confidence interval for the odds ratio with its upper and lower limits.

Table 5 shows the results of Binary Logistic Regression analysis. In the MCI group, people who have the habit of drinking red wine (compared with those who have no habit of drinking red wine) have lower risk of developing AD (OR = 0.463, 95%CI, 0.296–0.724, *p* = 0.001). After adjusting for age, education, dieting to lose weight, constipation, allergic history, cognitive-promoting drug use, diabetes in model b, this relationship is still statistically significant (OR = 0.503, 95%CI, 0.302–0.837, *p* = 0.008). On the basis of model b, further correction of drinking frequency, consumption of fruit, like berries and APOE genotype carrying factors in model c did not change the results (OR = 0.296, 95%CI, 0.147–0.598, *p* = 0.001).

**Table 5:** Multivariate analysis of red wine consumption.

Group		Model	Reference group		B	SE	Wald	*p*	OR	95%CI
The MCI group and the AD group	Red wine									
		a	NO	YES	−0.769	0.228	11.398	0.001	0.463	0.296–0.724
		b	NO	YES	−0.688	0.260	6.985	0.008	0.503	0.302–0.837
		c	NO	YES	−1.217	0.359	11.514	0.001	0.296	0.147–0.598

a, basic model, no adjustment. b, adjusted for age, education, dieting to lose weight, constipation, allergic history, cognitive-promoting drug use, diabetes. c, adjusted for all variables in model b + drinking frequency, consumption of fruit, like berries, APOE genotype. B, Coefficient for the constant; SE, Standard error around the coefficient for the constant; p, p-value; OR, Exp (B); 95%CI, Confidence interval for the odds ratio with its upper and lower limits.

## 3 Discussion

This cross-sectional study explored the correlation between fruit consumption, drinking habits and different cognitive states of the middle-aged and elderly population. Our research show that compared with those who did not like berries, those who liked berries in the objective cognitive unimpaired group had a lower risk of cognitive impairment. We also found compared with those who ate <100grams grapes per day, those who had daily grape intake of 100-200grams in the objective cognitive unimpaired group had a lower risk of cognitive impairment. In addition, we found that compared with those who had no habit of drinking red wine, those who had a habit of drinking red wine in the MCI group had a lower risk of AD.

Similar to our findings, a study found that a single dose of flavonoid-rich blueberries may have a potential protective effect on the cognitive function of healthy elderly people (15). A randomized controlled trial found that eating wild blueberries had significant cognitive benefits for middle-aged people with cognitive health (16). Recent studies have found that wild blueberries can improve the cognitive ability of healthy elderly men and women (17). Supplementing berries can produce a variety of health benefits, such as reducing inflammation and oxidative stress, enhancing metabolic function, improving vascular function and enhancing nerve signal conduction (18). The study found that adding blueberries to the diet of middle-aged people may reduce the risk of dementia in later years, and middle-aged people who drank blueberries showed higher levels of mitochondrial uncoupling, which is related to longer life span and reduced oxidative stress (19). In addition, blueberry supplementation can enhance metabolism and reduce insulin resistance (19).

The main types of polyphenols in berries flavonoids including flavane-3-alcohol, flavanol and anthocyanin (20). Among them, anthocyanins are the main flavonoids in berries. Anthocyanins are a class of water-soluble flavonoids, possess the ability to act as free radical scavengers against harmful oxidants such as reactive oxygen and nitrogen species (20). The health benefits of anthocyanins have been widely defined, especially in the prevention of oxidative stress-related diseases such as neurodegenerative diseases (21). Anthocyanins can effectively reduce the neurotoxicity of amyloid β *in vivo* and *in vitro*, thus inhibit the cascade of apoptosis and protect neurons from injury (22). In addition, there is evidence that the preventive and therapeutic effects of anthocyanins on neurodegenerative diseases may be achieved by regulating intestinal flora and some anthocyanin metabolites such as protocatechuic acid, vanillic acid (23). Our study has not yet found the effect of eating berries on the cognitive function of people who already have cognitive impairment. However, some studies have found that anthocyanin-rich blueberry extract and anthocyanin metabolite protocatechuic acid can induce autophagy of neurons in aging mice by promoting autophagy-lysosomal pathway *in vivo* and *in vitro* models of mice with AD to reduce neuronal damage (24). Similarly, population studies have shown that anthocyanin-rich blueberry extract significantly improves brain function in older people with mild cognitive impairment, reduces the risk of dementia, and delays the progression of AD (25, 26).

The relationship between alcohol consumption and the risk of dementia is controversial. In a recent systematic review of meta-analysis, mild to moderate alcohol consumption had a protective effect on dementia (27). A recent national cohort study in South Korea showed that persistent light and moderate drinkers reduced the risk of all-cause dementia, while persistent heavy drinkers increased the risk of all-cause dementia (28). At the same time, the study also found that heavy drinkers reduced their alcohol intake to moderate levels and non-drinkers alcohol intake to mild levels had a lower risk of developing all-cause dementia (28). This study has not found that red wine is a protective factor in people with normal cognition, but it is a protective factor in people with MCI. We speculate that the possible reason is related to the dosage of drinking red wine. However, the above research only explores the correlation between alcohol consumption and dementia, but does not explore the correlation between drinking types and dementia.

Our study found that people with MCI who drink red wine can reduce the risk of AD, and those who do not suffer from objective cognitive impairment can reduce the risk of cognitive impairment by consuming 100-200 g grapes per day. The protective effect of grapes and grape wine on cognition may be related to the following mechanisms. Polyphenols are bioactive compounds that extensively existed in plant foods and have many health-promoting effects by the different mechanisms, such as antioxidation, anti-inflammation, immunomodulation, and modulating gut microbiota. Resveratrol is a natural phenolic compound and has been found in many foods. Many studies have found that grape and its fresh derivative grape juice have protective effects on the cognitive decline of human beings, and its effects may be related to polyphenols, especially resveratrol (29). Red wine is rich in many kinds of polyphenols, among which resveratrol is the main one. The antiaging mechanisms of resveratrol were mainly ameliorating oxidative stress, relieving inflammatory reaction, improving mitochondrial function, andregulating apoptosis (30–33). Resveratrol can promote the proteasome degradation of hyperphosphorylated tau protein and reduce the aggregation of tau protein, thus protecting neurons (30). Resveratrol is a natural antioxidant, which can protect nerves through its antioxidant and anti-inflammatory activities (31), then improve cerebrovascular function and reduce the risk of dementia (32). It was found that the mixture of rifampicin and resveratrol could significantly improve the cognitive function of mice, inhibit the accumulation of oligomer and restore the level of synaptophysin, that is, the level of presynaptic protein that promotes synaptic formation (33). Synaptic dysfunction is considered to be the pathological change of AD. In addition, the increased expression of brain-derived neurotrophic factor (BDNF) in the hippocampus was observed after the administration of resveratrol or mixture, which improved the cognitive function of mice (33).

## 4 Conclusion

The results of this study show that the daily grape intake of 100-200grams and the preference for berries are the protective factors of cognitive function in those with objective cognitive unimpaired. In addition, we also find that the habit of drinking red wine is the protective factor of cognitive function in people with MCI.

The innovation of this study is that the SCD group is introduced into the cognitive state grouping of the middle-aged and elderly, so that the cognitive state is divided in more detail. And we explored the relationship between the types of drinking, fruit intake habits and different cognitive states of middle-aged and elderly people in China. In addition, the study included more than 1,000 participants, who underwent detailed dietary habits questionnaires and in-depth neuropsychological evaluation.

The deficiency of this study is that factors such as soil quality, climate conditions, amount of sunlight, and cultivation methods can influence the quantity and variety of functional components in fruits. Therefore, even for the same type of fruit, differences in the quantity of functional components may exist among fruits grown in different regions. And it is a cross-sectional study, so we can not make causal inference. Besides this study did not combine with biomarkers for analysis. In the future, dietary intervention surveys based on good control should be conducted, preferably in clinical trials, where appropriate berries, alcohol and different dose schemes should be selected more carefully to determine accurate doses.

## Data availability statement

The datasets presented in this article are not publicly available due to confidentiality issues. Requests to access the datasets should be directed to corresponding authors, QG and LH.

## Ethics statement

The studies involving humans were approved by the Ethics Committee of the Shanghai Sixth People’s Hospital Affiliated to Shanghai Jiao Tong University School of Medicine (China). The studies were conducted in accordance with the local legislation and institutional requirements. The participants provided their written informed consent to participate in this study. Written informed consent was obtained from the individual(s) for the publication of any potentially identifiable images or data included in this article.

## Author contributions

XJ: Data curation, Validation, Writing – original draft, Writing – review & editing. MC: Data curation, Formal analysis, Investigation, Writing – review & editing. LC: Formal analysis, Investigation, Methodology, Writing – review & editing. QG: Funding acquisition, Project administration, Resources, Supervision, Writing – review & editing. LH: Data curation, Investigation, Methodology, Project administration, Writing – review & editing.
